# Effect of isometric exercises on the masseter muscle in older adults with missing dentition: a randomized controlled trial

**DOI:** 10.1038/s41598-021-86807-w

**Published:** 2021-03-31

**Authors:** Satoru Takano, Kohei Yamaguchi, Kazuharu Nakagawa, Kanako Yoshimi, Ayako Nakane, Takuma Okumura, Haruka Tohara

**Affiliations:** Department of Dysphagia Rehabilitation, Tokyo Medical and Dental University(TMDU), 1-5-45 Yushima, Bunkyo-ku, Tokyo, 113-8510 Japan

**Keywords:** Dentistry, Nutrition

## Abstract

Maintaining oral function in older individuals with missing teeth is important for leading a healthy and independent life. This study aimed to evaluate whether simple isometric exercises can maintain and improve the oral function [maximum occlusal force (MOF) and masticatory ability (MA)] and the masticatory muscle properties [masseter muscle thickness (MMT) and echo intensity (MMEI)] in older adults during the maintenance phase of removable prosthetic treatment. Participants were randomly categorized into the intervention and control groups. The mouthpieces were distributed, and participants were instructed to use them for exercising. The intervention group was instructed to perform maximum clenching for 10 s, whereas the control group was instructed to tap the teeth at an arbitrary speed for 10 s. Both were repeated five times at an interval of 5 s between each activity and twice daily for 4 weeks. The outcomes were measured after a month of exercise. The intervention group showed a significant improvement in the MOF, MMT during contraction, MMT at rest and MMEI during contraction. There were no significant differences in the MA and MMEI at rest. In the control group, no improvement was observed in any of the parameters. When the isometric exercises were performed using a mouthpiece, there was an improvement in the oral function and masseter muscle properties in older individuals with Eichner B status who used dentures.

## Introduction

### Background and objective

A decrease in the oral function, represented by the maximum occlusal force (MOF) and the masticatory ability (MA), is a risk factor for adverse events such as sarcopenia and death^1^. MOF and MA greatly influence dietary choices^2^; a reduced bite force quotient leads to protein, fiber, mineral, and vitamin deficiencies^3–5^, resulting in a risk of undernutrition^6^. Maintaining the oral function is important for maintaining a healthy and independent life in older individuals. Aging and tooth loss are mainly considered to be responsible for a decrease in the MA and MOF^7^; however, these are reportedly related to the properties of masticatory muscles, especially of the masseter (for e.g., quantity and quality). Masseter muscle thickness (MMT) is reportedly associated with MOF^8^, and masseter muscle echo intensity (MMEI) is negatively correlated with MOF and MMT^9^. Muscle echo intensity can be evaluated by an ultrasonic diagnostic device that identifies non-contractile tissues (such as fat and fibrous tissues) in the muscle, and indicates muscle quality^10^. Several previous studies have reported that the MA, MOF, and MMT are greatly improved with dental prosthetic treatments such as implants and dentures^11–13^. In addition to prosthetic treatment, isometric exercise is also used as a modality for maintaining oral function. A retrospective comparative study of 28 young people showed that a simple isometric exercise using a mouthpiece resulted in an improvement in the MOF^14^. However, there are no reports on an effective training for maintaining the oral function in older adults with a missing dentition. Therefore, the purpose of this study was to clarify whether simple isometric exercises can maintain and improve the oral function (MOF and MA) and masticatory muscle properties (MMT and MMEI) in older adults during the maintenance phase of removable prosthetic treatment.

## Methods

### Trial design

**Figure 1:**
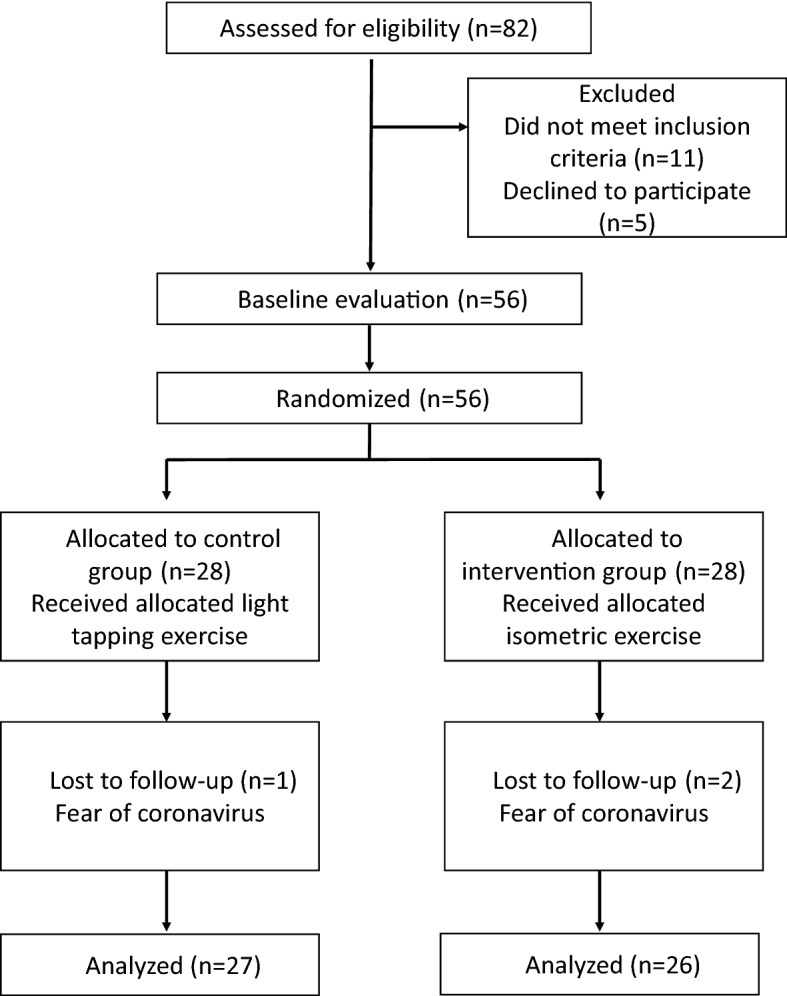
Flow diagram of this randomized-controlled trial.

This study was a multi-center, double-blinded, randomized, controlled, parallel clinical trial. Figure 1 shows the flow diagram of this randomized, controlled trial. This study was performed with the approval of the Clinical Research Ethics Committee of the Tokyo Medical and Dental University (D2018-021), and was registered in the UMIN clinical trial registration system on August 08, 2018 (UMIN000032933). This study was also conducted in accordance with the latest revision of the Declaration of Helsinki.

### Participants

Participants were adults who were aged 65 years and above and had removable dentures as prostheses for partial edentulism. They were recruited at an outpatient clinic (Tokyo, Japan) and at the dental hospital of the Tokyo Medical and Dental University.

The state of tooth-loss was categorized using the Eichner classification^15^. The inclusion criteria were as follows: (1) Eichner B-1 to B-3 groups that have occlusal support areas, which are the upper, lower, left, and right premolars and molars, and one to three of these are lost and (2) patients in the maintenance phase who had already completed denture adjustment. Patients excluded from the study were those who: (1) had difficulty in following instructions, (2) had progressive neuromuscular disease, (3) had severe temporomandibular joint symptoms (for e.g., trismus and pain during jaw movement), (4) required denture adjustment (for e.g., pain due to the denture), (5) had mobile teeth due to severe periodontal disease, and (6) had parafunctional findings. All participants had used the current dentures for at least 4 weeks. Written informed consent was obtained from all participants after a detailed explanation of the procedure.

### Intervention

**Figure 2:**
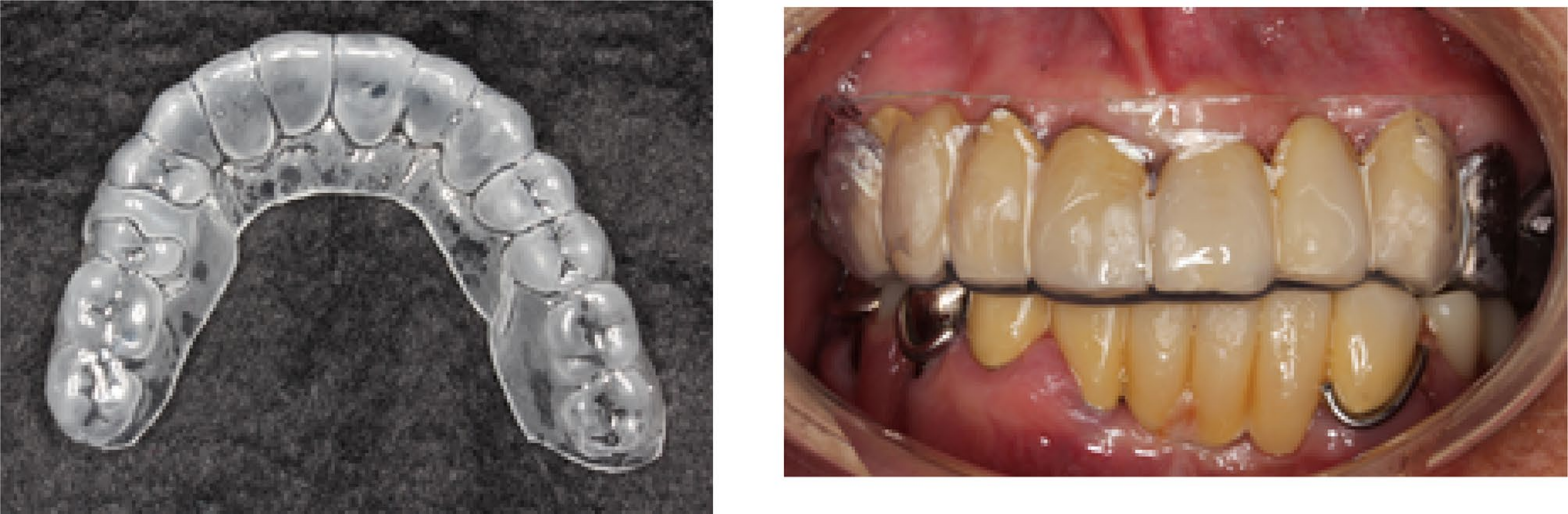
Mouthpiece and its placement in the oral cavity.

At the beginning of the study, the baseline outcomes were measured, and the impression of the upper jaw was obtained for the mouthpiece. The mouthpiece was fabricated by softening a 1.0-mm-thick thermoplastic sheet (for soft splint), which was then adapted on a plaster model with the help of a vacuum adapter (Yamahachi Dental Industry, Aichi, Japan), as described in a previous study^14^. On the next visit, the participants received the mouthpieces after proper adjustment and were randomly divided into two groups (Fig. 2). Thereafter, one of the dentists (who is not the doctor who measured the baseline outcomes) explained the intervention method to the participants.

### Intervention group

In the intervention group, a denture was provided to replace missing teeth in either the upper or lower jaw, and then a mouthpiece was fabricated for the upper jaw. If there was a denture in the upper jaw, the mouthpiece was worn over the denture. Subsequently, the participant was instructed to perform maximum clenching for 10 s. The training was repeated five times at an interval of 5 s between each clenching activity.

### Control group

In the control group, a denture was provided to replace missing teeth in either the upper or lower jaw, and a mouthpiece was then fabricated for the upper jaw. If there was a denture in the upper jaw, the mouthpiece was worn over the denture. However, unlike in the intervention group, participants in the control group were instructed to tap their teeth at an arbitrary speed for 10 s, and this was repeated five times at an interval of 5 s.

Both groups performed their respective exercises twice a day, in the morning and evening, for a period of 4 weeks.

### Measurement of the MOF, MA, MMT, and MMEI

The MOF, MA, MMT, and MMEI were measured using appropriate diagnostic devices. Participants were instructed to be seated on chairs in a relaxed position with their backs stretched, feet firmly touching the floor, and the Frankfurt horizontal plane parallel to the floor.

To measure the MOF, a pressure-sensitive film (Dental Prescale II, GC, Tokyo, Japan) was aligned with the dentition, and the participant was instructed to bite with maximum force. The scanner was calibrated^16^, the pressure-sensitive film was fitted to the template, and analysis was performed using an analysis software (Bite Force Analyzer GC, Tokyo, Japan).

For the measurement of MA, participants were instructed to freely chew a cylindrical-shaped gummy jelly (GC, Tokyo, Japan) composed of 40% maltose, 10% sorbitol, and 5% glucose, for 20 s. After chewing, the participants were asked to hold 10 mL of distilled water in their mouth and to spit the gummy jelly, distilled water, and saliva into a cup with a filter. The glucose concentration in the filtrate was measured using a glucose measuring device (Glucosensor GS-II, GC, Tokyo, Japan)^17^.

An ultrasonic diagnostic device (fST9600 Lequio Power Technology, Naha, Japan) was used to measure the MMT and MMEI. A linear probe was used with a broadband frequency ranging from 6.7 to 8.0 MHz. Scanning was performed in the B mode. The scanning depth was 38 mm and the gain was 80 dB, and these values were constant during the measurement. The measurements were performed by a dentist with 12 years of clinical experience.

As described in a previous study^18^, the probe was placed parallel to the mandibular margin, approximately midway between the zygomatic arch and the mandibular angle, and perpendicular to the skin surface. In accordance with existing literature, the probe was applied lightly^19^.

While measuring the thickness of the masseter muscle and its echo intensity, the patients were instructed to maintain the mandibular rest position for at least 20 min before ultrasonography in order to reduce the effects of muscle contraction on the blood flow and interstitial fluid^20^.

**Figure 3:**
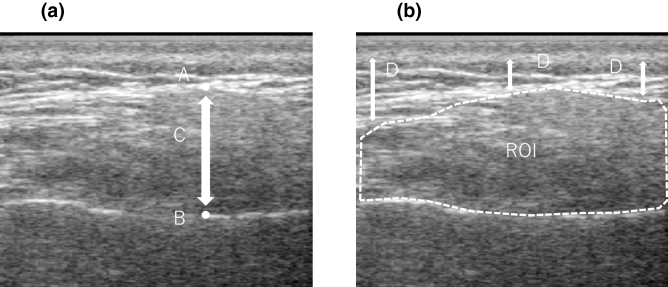
Masseter muscle evaluation by ultrasound diagnostic apparatus. **(a)** Masseter muscle thickness (MMT). **(b)** Masseter muscle echo intensity (MMEI) measurement. (A) Masseter muscle surface (B) Mandibular ramus (C) Masseter muscle thickness (D) subcutaneous fat thickness. *ROI* : region of interest.

Ultrasound images were analyzed using the Image-J software version 1.49 (National Institutes of Health, Bethesda, MD), and the maximum distance from the exterior portion of the ramus to the masseteric fascia was measured as MMT (Fig. 3). When measuring the MMEI, the region of interest was set to include the entire masseter muscle (Fig. 3). Echo intensity is attenuated due to the influence of subcutaneous fat; therefore, the following correction calculation was performed:

$${\text{Corrected echo intensity}} = {\text{uncorrected echo intensity}} \,+ \,{\text{subcutaneous fat thickness }}\left[ {{\text{cm}}} \right] \,\times\, {4}0.{5278}.$$

The corrected masseter muscle echo intensity was used as the measured value^10,21^. Subcutaneous fat thickness was measured at three locations and the average of the three measurements was used for calculations.

### Sample size

G * Power software version 3.1 (Kiel University) was used to calculate the sample size. The effect size was set to 0.8 in accordance with previous studies^11,14^ (d = 0.65, 0.88)^22^. When the α value was set to 0.05, the detection power was set to 0.8 and the effect size d to 0.8, and the required number of samples was 52. The number of dropouts was expected to be 8% for a total of 56 subjects.

### Randomization

The sampling followed a randomized stratified block method that combined a stratified method (gender) and a block method (intervention, control). One dentist was in charge of the outcome measurement, while the participants who received the dentures and mouthpieces were trained by another dentist. In other words, the dentist who measured the outcome and the dentists who explained the allocation and intervention methods were different. Therefore, both the research participants and the dentists who measured the outcomes were blinded.

### Statistical method

The Shapiro–Wilk test was performed for each measured value to confirm its normality.

Parametric (MA, rMMT, cMMT, rMMEI, cMMEI) and nonparametric (MOF) items were compared between the two groups using the two-sampled *t* test and the Mann–Whitney *U* test, respectively, before and after the intervention.

For each group comparison before and after the intervention, a paired *t*-test was used for parametric items and the Wilcoxon signed-rank test was used for nonparametric items.

A p value of <0.05 was considered statistically significant.

## Results

### Participants

Research participants were recruited from January 2019 to September 2020. Fifty-six participants were randomly assigned to two groups. Three dropped out of the study due to anxiety regarding hospital visits during the ongoing COVID-19 pandemic, and the final number of participants was 53 (Fig. 1). Table 1 shows the participants’ background characteristics in both groups.

**Table 1:** Baseline characteristics of the participants (n = 53).

	Group		*P* value
	Control (n = 27)	Intervention (n = 26)	
Age, years, median(IQR)	76.00 (10)	73.27 (8)	
**Gender**			0.908^b^
Male	16	15	
Female	11	11	
Body height, cm, mean(SD)	156.52 (± 6.45)	159.15 (± 7.51)	0.176^c^
Body weight, kg, mean(SD)	53.98 (± 9.85)	59.10 (± 8.87)	0.048*^,c^
BMI, kg/m^2^, mean(SD)	21.93 (± 3.23)	23.33 (± 2.82)	0.099^c^
**Eichner**			0.280^b^
B1	6	6	
B2	6	10	
B3	15	10	
MA, mg/dL, mean (SD)	181.19 (± 54.87)	178.54 (± 62.81)	0.871^c^
MOF, N, median (IQR)	539.00 (553.1)	608.45 (434.5)	0.176^a^
rMMT, mm, mean (SD)	7.87 (± 1.73)	8.89 (± 2.16)	0.064^c^
cMMT, mm (SD)	10.33 (± 2.46)	11.53 (± 2.61)	0.088^c^
rMMEI, mean (SD)	158.25 (± 17.89)	149.09 (± 17.20)	0.063^c^
cMMEI, mean (SD)	135.39 (± 20.93)	131.49 (± 16.16)	0.452^c^

*IQR* interquartile range, *SD* standard deviation, *BMI* body mass index, *MA* masticatory ability, *MOF* maximum occlusal force, *rMMT* masseter muscle thickness at rest, *cMMT* masseter muscle thickness during contraction, *rMMEI* masseter muscle echo intensity at rest, *cMMEI* masseter muscle echo intensity during contraction.

*p-value < 0.05.

^a^Mann–whitney *U* test.

^b^Pearson’s χ^2^ test.

^c^Two sample *t* test.

### Outcomes

At the baseline measurement stage, no significant differences were observed in any items, except for the body weight (Table 1). After the intervention, the intervention group showed a significantly greater improvement in the MOF (P = 0.001), MMT during contraction (cMMT) (P = 0.017), MMT at rest (rMMT) (P = 0.017), and MMEI during contraction (cMMEI) (P = 0.042) as compared to the control group. There was no significant difference in the MA and MMEI at rest (rMMEI) between the two groups (Table 2).

**Table 2:** Comparison between the control (n = 27) and intervention (n = 26) groups before and after intervention.

	Baseline		*p* value	At 4 weeks post intervention		*p* value
	Control (n = 27)	Intervention (n = 26)		Control (n = 27)	Intervention (n = 26)	
MA, mg/dL, mean (SD)	181.19 (± 54.87)	178.54 (± 62.81)	0.871^a^	185.19 (± 60.92)	197.92(± 52.89)	0.421^a^
MOF, N, median (IQR)	539.00 (553.1)	608.45 (434.5)	0.176^b^	564.80(511.6)	905.15(525.4)	0.001*^,b^
rMMT, mm, mean (SD)	7.87 (± 1.73)	8.89 (± 2.16)	0.064^a^	7.69 (± 1.92)	9.07 (± 2.16)	0.017*^,a^
cMMT, mm, mean (SD)	10.33 (± 2.46)	11.53 (± 2.61)	0.088^a^	10.42 (± 2.68)	12.65 (± 2.76)	0.017*^,a^
rMMEI, mean (SD)	158.25(± 17.89)	149.09(± 17.20)	0.063^a^	156.30(± 17.62)	149.28(± 18.02)	0.158^a^
cMMEI, mean (SD)	135.39(± 20.93)	131.49(± 16.16)	0.452^a^	134.59(± 18.23)	124.72(± 16.04)	0.042*^,a^

*SD* standard deviation, *IQR* interquartile range, *MA* masticatory ability, *MOF* maximum occlusal force, *rMMT* masseter muscle thickness at rest, *cMMT* masseter muscle thickness during contraction, *rMMEI* masseter muscle echo intensity at rest, *cMMEI* masseter muscle echo intensity during contraction.

*p-value < 0.05.

^a^Two-sampled *t* test.

^b^Mann–Whitney *U* test.

Significant improvements in the MOF (P < 0.001), cMMT (P = 0.003), cMMEI (P = 0.007), and MA (P = 0.007) were observed in the intervention group following intervention. In the control group, no improvement was observed in any of the measured values (Table 3).

**Table 3:** Intra-group comparison in the control (n = 27) intervention (n = 26) groups before and after intervention.

	Control		*p* value	Intervention		*p* value
	Baseline	Post intervention		Baseline	Post intervention	
MA, mg/dL, mean(SD)	181.19 (± 54.87)	185.19 (± 60.92)	0.549^a^	178.54 (± 62.81)	197.92 (± 52.89)	0.007*^,a^
MOF, N, median(IQR)	539.00 (553.1)	564.80 (511.6)	0.683^b^	608.45 (434.5)	905.15 (525.4)	0.000*^,b^
rMMT, mm, mean(SD)	7.87 (± 1.73)	7.69 (± 1.92)	0.280^a^	8.89 (± 2.16)	9.07 (± 2.16)	0.220^a^
cMMT, mm, mean(SD)	10.33 (± 2.46)	10.42 (± 2.68)	0.673^a^	11.53 (± 2.61)	12.65 (± 2.76)	0.003*^,a^
rMMEI, mean(SD)	158.25 (± 17.89)	156.30 (± 17.62)	0.373^a^	149.09(± 17.20)	149.28(± 18.02)	0.940^a^
cMMEI, mean(SD)	135.39 (± 20.93)	134.59 (± 18.23)	0.789^a^	131.49(± 16.16)	124.72(± 16.04)	0.007*^,a^

*SD* standard deviation, *IQR* interquartile range, *MA* masticatory ability, *MOF* maximum occlusal force, *rMMT* masseter muscle thickness at rest, *cMMT* masseter muscle thickness during contraction, *rMMEI* masseter muscle echo intensity at rest, *cMMEI* masseter muscle echo intensity during contraction.

*p-value < 0.05.

^a^Paired *t* test.

^b^Wilcoxon signed-rank test.

## Discussion

In this intervention study, the following observations were made: On performing isometric exercises using a mouthpiece, there was (1) an improvement in the oral function and (2) an improvement in the masseter muscle properties in older individuals with an Eichner B status who used dentures.

First, as a result of training, the oral function, especially MOF, improved. There are many reports on resistance training for limb muscles, which increases the muscle mass and strength^23,24^. It has been reported that a significant improvement in the MOF was achieved by resistance training in adults without missing teeth^14^. The masseter tissue is dominated by Type 1 muscle fibers and has a composition different from that of the quadriceps femoris, which has a large number of Type 2 muscle fibers^25^. Type 1 muscle fibers are more susceptible to disuse than to aging^26^. Tooth loss is also an independent factor associated with the MMT^27^. Furthermore, it has been reported that chewing exercises for older people with 24 or more remaining teeth improved the MOF^28^. In this study, we targeted older adults with missing teeth and found an improvement in the MOF, which was consistent with the findings of previous studies^14,28^.

The pre- and post-intervention MA differed significantly in the intervention group, but not in the control group. Since the masticatory ability and occlusal force are related^29^, improvement of occlusal force by isometric exercise may contribute to the improvement of the masticatory ability. However, the masticatory ability is also an important factor associated with the tongue function^30^, therefore the lack of significant difference in MA could be probably due to the target muscle of the training being the masseter, and not the tongue.

Second, an improvement in the masseter muscle properties was also observed. Significant improvements were observed in both the rMMT and the cMMT. It has been found that age-related changes strongly affect the muscle strength and muscle mass^31^. Muscle weakness is a risk factor for subsequent hospitalization and death^32^. Muscle strengthening is known to occur before muscle mass gain^33^. While muscle hypertrophy was said to occur after 6 weeks^34^, it has recently been reported to occur in the skeletal muscle (for e.g., the thigh muscle) from about 4 weeks^35^. In this study as well, a significant improvement in masseter muscle hypertrophy was observed at 4 weeks. One of the factors that causes muscle hypertrophy at a relatively early stage is the high sensitivity of the masseter muscle to testosterone. Testosterone promotes protein synthesis in the muscle^36^. In a study of rats, injection of testosterone increased the masseter muscle mass by 38%, which was reportedly more sensitive than other muscles^37^. Isometric exercise has been shown to significantly increase the testosterone levels^38,39^. The masseter may be a muscle that is more affected by isometric exercise as compared to the other muscles.

In this study, the cMMEI was also significantly improved. The higher the number, the more non-contractile tissues in the muscle, indicating a decrease in muscle quality. Previous studies in healthy older adults have shown a strong negative correlation between the cMMT and cMMEI^9^. It is considered that an improvement in the cMMT by isometric exercise also led to an improvement in the cMMEI.

This study has several limitations. First, it targeted older people in the Eichner B group with a limited number of missing teeth. In order to clarify the usefulness of isometric exercises for the masseter muscle, it will be necessary to conduct studies on patients with multiple missing teeth, including the Eichner C group. Second, the training period for this study was 1 month, and long-term effects were not considered. Tracking the long-term effects of isometric exercises is warranted in future research. Third, echo is used for muscle mass evaluation. It has been reported that there is a strong correlation between the muscle cross section in magnetic resonance imaging and the muscle thickness estimated in the B mode of echo at the same location; however, the gold standard for muscle mass evaluation is magnetic resonance imaging and computed tomography.

## Significance and future implications

In this study, isometric exercise using a mouthpiece was shown to significantly improve occlusal force and masseter muscle properties (quantity and quality) in older people using Eichner B dentures. Masseter muscle hypertrophy was confirmed in a short span of 1 month. Therefore, it is possible to sufficiently improve and maintain oral function even in older individuals who are in the maintenance phase after the completion of prosthetic treatment. The possibility of self-management of oral function and masticatory muscle properties has been demonstrated in this study. This time, we have achieved good results in the research targeting the Eichner B group, and we would like to perform further studies targeting the C group in the future.

## References

[1] Cruz-Jentoft AJ, Sayer AA (2019). Sarcopenia. Lancet.

[2] Nowjack-Raymer RE, Sheiham A (2003). Association of edentulism and diet and nutrition in US adults. J. Dent. Res..

[3] Borie E (2014). Maximum bite force in elderly indigenous and non-indigenous denture wearers. Acta Odontol. Latinoam..

[4] Moynihan P (2009). Researching the impact of oral health on diet and nutritional status: methodological issues. J. Dent..

[5] Nowjack-Raymer RE, Sheiham A (2007). Numbers of natural teeth, diet, and nutritional status in US adults. J. Dent. Res..

[6] Houston, D. K. *et al*. Dietary protein intake is associated with lean mass change in older, community-dwelling adults: the health, aging, and body composition (Health ABC) study. *Am. J. Clin. Nutr.* **87**1, 50e5 (2008). 10.1093/ajcn/87.1.150 18175749

[7] Okamoto, N., Amano, N., Nakamura, T. & Yanagi, M. Relationship between tooth loss, low masticatory ability, and nutritional indices in the elderly: a cross-sectional study. *B.M.C. Oral Health. 19*, 110 (2019). 10.1186/s12903-019-0778-5 PMC6567659 31196057

[8] Raadsheer MC, van Eijden TM, van Ginkel FC, Prahl-Andersen B (1999). Contribution of jaw muscle size and craniofacial morphology to human bite force magnitude. J. Dent. Res..

[9] Yamaguchi K (2019). Factors associated with masseter muscle quality assessed from ultrasonography in community-dwelling elderly individuals: A cross-sectional study. Arch. Gerontol. Geriatr..

[10] Young HJ, Jenkins NT, Zhao Q, Mccully KK (2015). Measurement of intramuscular fat by muscle echo intensity. Muscle Nerve.

[11] Melo ACM, Ledra IM, Vieira RA, Coró ER, Sartori IAM (2018). Maximum bite force of edentulous patients before and after dental implant rehabilitation: Long-term follow-up and facial type Influence. J. Prosthodont..

[12] Müller F (2013). Implant-supported mandibular overdentures in very old adults: A randomized controlled trial. J. Dent. Res..

[13] von der Gracht I, Derks A, Haselhuhn K, Wolfart S (2017). EMG correlations of edentulous patients with implant overdentures and fixed dental prostheses compared to conventional complete dentures and dentates: A systematic review and meta-analysis. Clin. Oral Implants Res..

[14] Thompson DJ, Throckmorton GS, Buschang PH (2001). The effects of isometric exercise on maximum voluntary bite forces and jaw muscle strength and endurance. J. Oral Rehab..

[15] Eichner K (1955). Über eine Gruppeneintelung des lückengebisses für die prothetik. Deutsche Zahnarztliche Z..

[16] Shiga H (2020). Comparison of two dental pre scale systems used for the measurement of occlusal force. Odontology.

[17] Takeshima T, Fujita Y, Maki K (2019). Factors associated with masticatory performance and swallowing threshold according to dental formula development. Arch. Oral Biol..

[18] Serra, M. D., Duarte Gavião, M. B. & dos Santos Uchôa, M. N. The use of ultrasound in the investigation of the muscles of mastication. *Ultrasound Med. Biol*. **34**, 1875–1884 (2008). 10.1016/j.ultrasmedbio.2008.05.009 18774217

[19] Thoirs K, English C (2009). Ultrasound measures of muscle thickness: Intra-examiner reliability and influence of body position. Clin. Physiol. Funct. Imaging..

[20] Yoshiko A (2017). Effect of 12-month resistance and endurance training on quality, quantity, and function of skeletal muscle in older adults requiring long-term care. Exp. Gerontol..

[21] Stock MS (2018). Echo intensity versus muscle function correlations in older adults are influenced by subcutaneous fat thickness. Ultrasound Med. Biol..

[22] Cohen J (1992). A power primer. Psycohol. Bull..

[23] Wang E (2017). Impact of maximal strength training on work efficiency and muscle fiber type in the elderly: Implications for physical function and fall prevention. Exp. Gerontol..

[24] Abe T, DeHoyos DV, Pollock ML, Garzarella L (2000). Time course for strength and muscle thickness changes following upper and lower body resistance training in men and women. Eur. J. Appl. Physiol..

[25] Monemi M, Eriksson PO, Eriksson A, Thornell LE (1998). Adverse changes in fibre type composition of the human masseter versus biceps brachii muscle during aging. J. Neurol. Sci..

[26] Cullins MJ, Connor NP (2017). Alterations of intrinsic tongue muscle properties with aging. Muscle Nerve..

[27] Yamaguchi, K. *et al.* Relationship of aging, skeletal muscle mass, and tooth loss with masseter muscle thickness. *B.M.C. Geriatr.* **18**, 67 (2018). 10.1186/s12877-018-0753-z PMC5844127 29519234

[28] Kim MJ (2020). Effects of chewing exercises on the occlusal force and masseter muscle thickness in community-dwelling Koreans aged 65 years and older: A randomized assessor-blind trial. J. Oral Rehabil..

[29] Kosaka T (2018). Factors influencing the changes in masticatory performance: The Suita study. JDR Clin. Trans. Res..

[30] Sagawa K (2019). Tongue function is important for masticatory performance in the healthy elderly: A cross-sectional survey of community-dwelling elderly. J. Prosthodont. Res..

[31] Goodpaster BH (2006). The loss of skeletal muscle strength, mass, and quality in older adults: The health, aging and body composition study. J. Gerontol. A. Biol. Sci. Med. Sci..

[32] Fried, L. P. *et al*. Cardiovascular Health Study Collaborative Research Group. Frailty in older adults: evidence for a phenotype. *J. Gerontol. A. Biol. Sci. Med. Sci.* **56,** M146–M156 (2001). 10.1093/gerona/56.3.m146 11253156

[33] Moritani T, DeVries HA (1979). Neural factors versus hypertrophy in the time course of muscle strength gain. Am. J. Phys. Med..

[34] Staron RS (1994). Skeletal muscle adaptations during early phase of heavy-resistance training in men and women. J. Appl. Physiol..

[35] DeFreitas, J. M., Beck, T. W., Stock, M.S., Dillon, M.A. & Kasishke, P. R. 2nd. An examination of the time course of training-induced skeletal muscle hypertrophy. *Eur. J. Appl. Physiol*. **111**, 2785–2790 (2011). 10.1007/s00421-011-1905-4 21409401

[36] Griggs RC (1989). Effect of testosterone on muscle mass and muscle protein synthesis. J. Appl. Physiol..

[37] Widmer CG, Morris-Wiman J (2010). Limb, respiratory, and masticatory muscle compartmentalization: Developmental and hormonal considerations. Prog. Brain Res..

[38] Kraemer WJ, Ratamess NA (2005). Hormonal responses and adaptations to resistance exercise and training. Sports Med..

[39] Vingren JL (2010). Testosterone physiology in resistance exercise and training: the up-stream regulatory elements. Sports Med..

