# Author Correction: Effect of isometric exercises on the masseter muscle in older adults with missing dentition: a randomized controlled trial

**DOI:** 10.1038/s41598-021-88765-9

**Published:** 2021-04-21

**Authors:** Satoru Takano, Kohei Yamaguchi, Kazuharu Nakagawa, Kanako Yoshimi, Ayako Nakane, Takuma Okumura, Haruka Tohara

**Affiliations:** Department of Dysphagia Rehabilitation, Tokyo Medical and Dental University(TMDU), 1-5-45 Yushima, Bunkyo-ku, Tokyo, 113-8510 Japan

Correction to: *Scientific Reports* 10.1038/s41598-021-86807-w, published online 31 March 2021

The original version of this Article contained errors in the spelling of the authors Satoru Takano, Kohei Yamaguchi, Kazuharu Nakagawa, Kanako Yoshimi, Ayako Nakane, Takuma Okumura & Haruka Tohara which were incorrectly given are S. Takano, Kohei Yamaguchi, K. Nakagawa, K. Yoshimi, A. Nakane, T. Okumura & H. Tohara respectively.

These errors have now been corrected in the PDF and HTML versions of the Article.

